# Cutis laxa associated with a missense variant in elastin gene

**DOI:** 10.1016/j.jdcr.2025.09.048

**Published:** 2025-10-27

**Authors:** Jay R. Patel, Vedha Vaddaraju, Jennifer Dykeman, Eran Tallis, Kathleen Mannava, Jinia El-Feghaly, Mary Gail Mecurio

**Affiliations:** a Department of Dermatology, University of Rochester Medical Center, Rochester, New York; b Division of Clinical Genetics, University of Rochester Medical Center, Rochester, New York

**Keywords:** connective tissue disorder, cutis laxa, elastic fibers, ELN gene, missense variant, vascular abnormalities

## Introduction

Cutis laxa is a rare connective tissue disorder characterized by loose, inelastic skin and systemic manifestations, including vascular abnormalities.^1^ Genetic mutations affecting elastin (ELN) and other extracellular matrix components contribute to its heterogeneity, making diagnosis and management complex.^1^ Although cutis laxa is typically associated with known pathogenic variants, we present a case of cutis laxa associated with a novel ELN variant initially brought forth by vascular complications.

## Case report

A 56-year-old man was evaluated for skin laxity and vascular tortuosity following a type B aortic dissection. He initially presented to the emergency department with hematuria after falling from a horse. Imaging revealed a type B aortic dissection classified as a tear in the descending aorta extending from the renal to iliac arteries, as well as sciatic artery tortuosity and aneurysmal dilation. Following a preoperative angiogram by vascular surgery, he had a left brachial artery pseudoaneurysm, which required surgical repair. Given the patient's vascular tortuosity and skin laxity noted on examination, genetics was consulted to identify a pathogenic mutation for this clinical presentation. Completion of a connective tissue disorders panel identified a novel, heterozygous missense c.484G>A variant in the ELN gene (p. Gly162Ser). This variant was classified as a variant of uncertain significance for the following reasons: this variant had not been reported in the literature in individuals affected with ELN-related conditions (autosomal dominant supravalvular aortic stenosis, autosomal dominant cutis laxa, and Williams syndrome),^2^, ^3^, ^4^, ^5^ presence in population databases (Genome Aggregation Database frequency of 0.006%), and an internal functional study, indicating that the variant is not expected to disrupt ELN protein function with a negative predictive value of 80%. Despite this, clinically, there was suspicion for ELN-related cutis laxa due to skin laxity, craniofacial characteristics, aortic dissection, and vascular tortuosity. Therefore, to clarify the pathogenicity of the reported variant, a referral to dermatology was placed.

**Fig 1:**
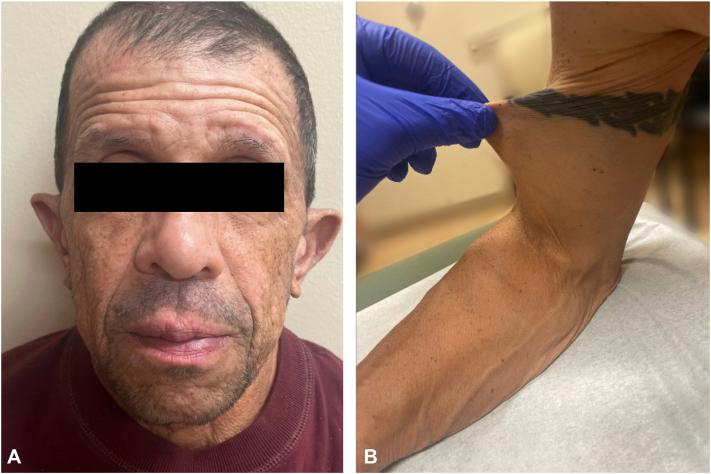
Clinical images of face and arm. **A,** Excessive skin laxity on the cheeks with notable facial features including low-set ears and accentuation of the nasolabial fold. **B,** Skin pull test on the upper portion of the arm reveals loose skin with decreased elasticity, consistent with cutis laxa.

**Fig 2:**
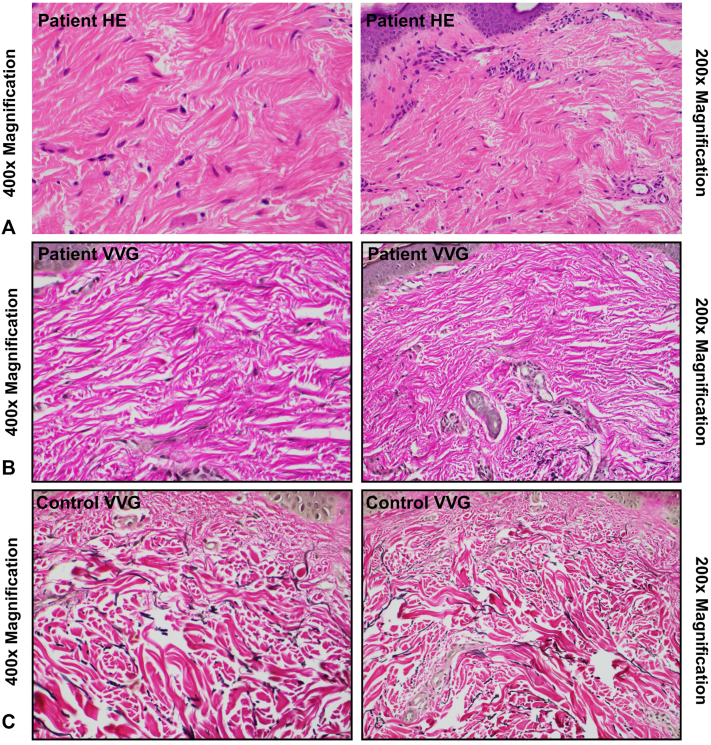
Histopathologic findings. **A,** Skin biopsy from the photo-protected upper portion of the arm showing mild vascular ectasia, stained using hematoxylin and eosin (HE). **B,** Skin biopsy from the photo-protected upper portion of the arm shows minimal dermal inflammation and a marked reduction in elastic fibers on Verhoeff–Van Gieson (VVG) staining, supporting a diagnosis of cutis laxa. **C,** For comparison, a skin biopsy from an age-matched control subject shows a normal and dense network of elastic fibers.

An outpatient dermatology referral elicited additional history, including an inguinal hernia repair at the age of 7 years and recurrent diverticulitis. Physical examination revealed loose, lax skin on the face, upper extremities, and arms, with low-set ears (Fig 1, *A*). Skin pull test identified loose skin with decreased elasticity (Fig 1, *B*). A skin biopsy from the photo-protected upper portion of the arm demonstrated mild vascular ectasia and marked reduction in elastic fibers, demonstrated by decreased Verhoeff–Van Gieson staining within the papillary dermis (Fig 2), supporting a diagnosis of cutis laxa. With this new diagnosis of cutis laxa, vascular surgery meticulously planned the repair of the patients' tortuous sciatic aneurysms and continued the patient on medical management for vascular complications with aspirin, apixaban, atorvastatin, amlodipine, and metoprolol.

## Discussion

The constellation of clinical, radiologic, genetic, and histopathologic findings supports a diagnosis of cutis laxa, representing a potential mild phenotype affecting both the integumentary and vascular systems associated with c.484G>A *ELN* variant. The detected p.Gly162Ser variant is considered pathogenic for the reason that the phenotype of the patient aligns very closely with the known spectrum of ELN-related disorders, and the genetic testing done identified no other causative variants. This case differs from typical autosomal dominant cutis laxa, which is caused exclusively by nonsense or frameshift mutations. We hypothesize that this mutation may lead to decreased translation and transcription of the ELN protein due to the absence of the fibers as seen on pathology. Despite this variation in mutation, the clinical phenotype of characteristic facial features, redundant skin, and arterial aneurysms overlaps.^1^ Vascular tortuosity most closely overlaps with both dominant *ELN* and recessive *FBLN4* variants.^1^ Given the heterogeneity of molecular pathogenesis in cutis laxa, this case underscores the critical role of a multidisciplinary approach in diagnosis and management. The discovery of this genetic variant adds to the existing heterogeneous group of cutis laxa syndromes requiring vascular surveillance.

## Conflicts of interest

None disclosed.
